# The association between waist circumference and adult asthma attack using nationally representative samples

**DOI:** 10.1186/s12889-024-18656-x

**Published:** 2024-04-25

**Authors:** Xiang Liu, Shuang Tian, Ting Zhao

**Affiliations:** 1 Emergency Intensive Care Unit, Qingdao Municipal Hospital, Qingdao, China; 2 Emergency Department, Qingdao Municipal Hospital, Qingdao, China; 3 Health Care Geriatrics Ward, Qingdao Municipal Hospital, Qingdao, China

**Keywords:** Waist circumference, Asthma attack, Asthma, Association analysis

## Abstract

**Aims:**

This study aims to explore the relationship between waist circumference and asthma attack in adults.

**Methods:**

In this cross-sectional study, we analysed data from 5,530 U.S. adults diagnosed with asthma. Participants were categorized into two groups based on their experience of asthma attacks: with or without asthma attacks. We employed adjusted weighted logistic regression models, weighted restricted cubic splines, subgroup and sensitivity analyses to assess the association between waist circumference and asthma attack.

**Results:**

The median age of all participants was 43 years, and the median waist circumference was 98.9 cm, with a median BMI was 28.50 kg/m^2^. Participants in the asthma attack group had significantly higher waist circumferences than those in the non-attack group (*P* < 0.001). After full adjustment for body mass index-defined obesity, age, gender, race, education levels, poverty income ratio levels, smoking status, and metabolic syndrome, every 5 cm increase in waist circumference exhibited a 1.06 times higher likelihood of asthma attack probability. The weighted restricted cubic spline analysis demonstrated an increased risk of asthma attacks with rising waist circumference. Subgroup analyses confirmed this relationship across various groups differentiated by gender, age, and smoking status. When applying a stricter definition of asthma attack, the weighted logistic regression models showed robust association between waist circumference and asthma attack.

**Conclusion:**

Waist circumference is an independent predictor of asthma attacks. Our findings underscore the importance of waist circumference measurement in evaluating the risk of asthma attacks.

## Introduction

Asthma, a prevalent chronic respiratory condition, is marked by symptoms such as recurrent wheezing, breathlessness, and coughing, alongside airway inflammation and obstruction [1]. The prevalence of asthma is on the rise across numerous developing countries, contributing to a growing economic burden [2]. The 2015 Global Burden of Disease Study reports that approximate 358 million individuals were affected by asthma globally, with an increase of 12.6% compared to 1990 [3].

Asthma attack, characterized by the sudden onset or aggravation of symptoms like wheezing, shortness of breath, coughing, and chest tightness, results from a decrease in expiratory airflow [1]. Asthma attack poses the most substantial risk for asthma-related mortality and chronic complications [4]. Various factors contribute to asthma exacerbation, including medications [5], obesity [6–8], asthma duration [9], smoking [7], socioeconomic status [10, 11], allergic rhinitis [12], among others [13]. Uncontrolled asthma can disrupt daily activities and impose limitations on physical, emotional, and social well-being [14]. The management of these modifiable risk factors could enhance asthma control and life quality [7]. Nevertheless, poor asthma control increases the likelihood of exacerbations [4], and despite ongoing effort, asthma control rate remained unsatisfied [15]. In the United States, approximately 12 million individuals experience an acute exacerbation of asthma, with 25% requiring hospitalization [16].

Obesity represents another significant worldwide public health concern, affecting about 30% of the global population [17]. In current clinical practice, Body Mass Index (BMI) is the predominant anthropometric measure for obesity, widely used to assess the risk of obesity-related conditions [18–20]. A positive association between BMI and asthma was confirmed [21], and the natural history of asthma control can be impacted by BMI [22]. Adults who were obese had a higher likelihood of reporting poor asthma control compared to individuals with BMI below 25 kg/m^2^ [6]. Importantly, the weight reduction in asthma patients can discontinue/reduce asthma medications [23] and significantly improve asthma outcomes [24]. However, BMI reflects only general obesity, regardless of variations in body fat distribution and muscle composition [25]. Abdominal obesity, also known as central obesity, is an excessive accumulation of abdominal fat and is linked with a higher prevalence of asthma [26–33]. In 2020, an expert consensus pointed out that waist circumference offers a more accurate evaluation of the metabolic risk of fat distribution and should be included in clinical testing as a routinely measured ‘vital sign’ [34]. A meta-analysis has demonstrated that abdominal adiposity (measured by waist circumference) exhibits a positive association with asthma, which is similar in males and females [28]. Understanding the risk factors and combining them with appropriate treatment can help formulate management strategies to reduce the chance of asthma attacks [35].

Nevertheless, the association between asthma attack and abdominal obesity remains unclear. Therefore, this study aims to elucidate the association between waist circumference and asthma attack based on national representative population.

## Methods

### Study population

National Health and Nutrition Examination Survey (NHANES) is a cross-sectional study conducted to evaluate the health and nutritional status of individuals within the United States (http://wwwn.cdc.gov/nchs/nhanes). Since 1999, the NHANES team has conducted a national survey every two years and collected demographic, physical examination, laboratory indicators, and questionnaire information. The multi-stage stratified cluster random sampling design was employed to ensure the representativeness of the samples.

**Fig. 1:**
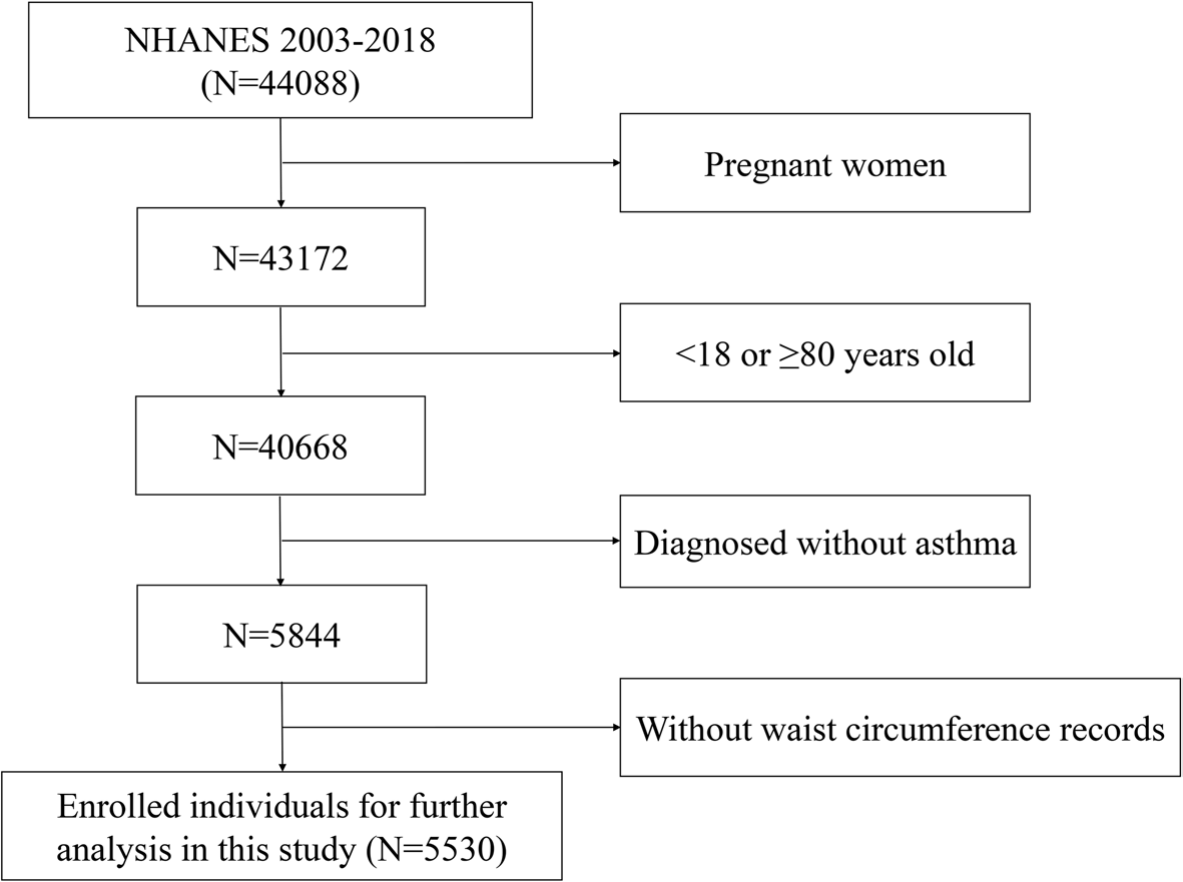
Flow chart of selection of participants from the National Health and Nutrition Examination Survey. *BMI: body mass index*

Figure 1 depicts the flow chart of the selection of eligible participants. This study included participants from 8 NHANES cycles over the period from 2003 to 2018. The following criteria were used for exclusion: 1) participants were pregnant women; 2) age < 18 or ≥ 80 years old; 3) participants without asthma; 4) missing data on waist circumference records. Finally, 5,530 individuals with asthma were included in this study. The Ethics Review Committee of the National Center has approved this study, with all participants signing a written informed consent.

### The definition of asthma and asthma attack

In this study, asthma was defined based on self-report or physician-diagnosed history obtained by questionnaires. Participants with a positive response to the question, ‘Has a doctor or other health professional ever told you that you have asthma?’, were considered as asthma patients [36].

The assessment of asthma attack was based on responses to the following two questions from the NHANES ‘Medical Conditions’ questionnaire [37]: 1) Regarding asthma attack in the past year: ‘During the past 12 months, have you had an episode of asthma or an asthma attack?’; and 2) Regarding emergency care visit for asthma in the past year: ‘During the past 12 months, have you had to visit an emergency room or urgent care center because of asthma?’ Consistent with a previous study on NHANES [38], participants meeting either criterion were identified as having experienced an asthma attack.

### Waist circumference and body mass index measurement

Height, weight, and waist circumference are measured by trained inspectors employing standard measuring instruments. The measurement of waist circumference involved direct assessment against the skin at the upper lateral edge of the iliac crests. The examiner positioned themselves on the right side of the participant, identified the right ilium of the pelvis by palpating the hip area, and demarcated a horizontal line above the highest lateral border. Additionally, waist circumference was divided into quartiles, with the lowest quartile (Q1) designated as the baseline group. BMI was assessed with subjects standing, wearing light clothing, and no shoes. BMI was calculated by dividing weight in kilograms by the square of the height in meters (kg/m^2^). The measurement was accurate to 0.1 cm. NHANES uses the apex of the right iliac crest as the measurement level. More detailed information is provided in the NHANES Questionnaire Protocol (http://wwwn.cdc.gov/nchs/nhanes).

### Covariates

Covariates such as demographic details, medical history, and health behavioural factors were incorporated to mitigate potential bias. Demographic information (age [39], gender [40, 41], race [42], and education), medical history [43–45], and health behavioural factors (smoking status [46, 47] and alcohol consumption [48]) were obtained from questionnaires.

From the demographic questionnaire, we collected continuous age data, as well as information on gender (male and female), ethnicity (non-Hispanic White, non-Hispanic Black, Mexican American, other Hispanic, and other races including Multi-Racial), and educational attainment (below high school, high school, and above high school). We used the poverty income ratio (PIR) as a measure of socioeconomic status, which reflects household income relative to the poverty threshold. The PIR values of < 1, 1–4, and ≥ 4 represent the low-, middle-, and high-income levels [49]. Information regarding self-reported diabetes history (yes/no), smoking status (never, former, and current smokers), and alcohol consumption (consuming at least 12 alcoholic drinks per year or not) was also acquired from the health questionnaire. Besides, metabolic syndrome is defined by a collection of metabolic risk factors, including: (1) fasting plasma glucose levels ≥ 100 mg/dL; (2) hypertension; (3) triglycerides ≥ 150 mg/dL; (4) high-density lipoprotein cholesterol levels < 40 mg/dL in men and < 50 mg/dL in women; and (5) a waist circumference exceeding 102 cm in men and 88 cm in women [50]. The presence of three or more of these risk factors qualifies for a diagnosis of metabolic syndrome [50]. Detailed information on study design and procedures is accessible on the NHANES website.

### Statistical analysis

We conducted weighted data analysis following the guidelines outlined in the NHANES analytic and reporting guidance document [51]. NHANES utilized an intricate survey framework to minimize the basis stemming from non-response, oversampling, and post-stratification. Every individual in the study was allocated a specific sampling weight, primary sampling unit (PSU), and stratum assignment, enabling the generation of representative nationwide projections. WTMEC2YR documents the survey weighting assigned to every participant who underwent body measurements at a Mobile Exam Center. Having analysed eight consecutive NHANES cycles, we computed the 16-year sample weight (WTMEC16YR) using the subsequent formula: WTMEC16YR = 1/8 × WTMEC2YR. In the subsequent statistical analysis, we incorporated survey weights to generate estimates that represent the population of the United States.

We reported that normally distributed continuous variables were provided as mean ± standard deviation and skewed distributed continuous variables were presented as median (Q1, Q3), decided by the weighted Kolmogorov–Smirnov test. Categorical variables were displayed as frequencies accompanied by their respective percentages. Baseline demographic characteristics were contrasted between participants with non- asthma attack and asthma attack using the one-way analysis of variance (normal distribution), the weighted Kruskal–Wallis test (skewed distribution), or the weighted chi-square test (categorical variables). Furthermore, we employed weighted logistic regression to assess the association between waist circumference and asthma attack, with subsequent calculation of odds ratios (ORs) and their corresponding 95% confidence intervals (CIs).

We conducted model adjustments accounting for BMI-defined obesity, age, gender, race/ethnicity, educational attainment, and smoking status. Furthermore, we depicted the links between waist circumference and asthma attack through the utilization of a weighted restricted cubic spline analysis, employing 5 knots positioned at the percentiles: 5%, 27.5%, 50%, 72.5%, and 95%. Subsequently, we conducted a subgroups analysis of logistic regression within distinct subgroups, encompassing categories such as gender (male or female), age (< 45 or ≥ 45 years), and smoking (not at all, some days or every day). Additionally, we performed sensitivity analysis by taking a stricter definition for asthma attack. Only those participants who both had an episode of asthma and had to visit an emergency room or urgent care centre due to asthma were identified with asthma attack. Statistical analysis was carried out using R software. A significance level of *P* < 0.05 was used to determine statistical significance.

## Results

### Characteristics of participants

Among the NHANES survey from 2003 to 2018, 5530 US adults were eventually included, of whom 2378 were male, and 3152 were female in this study. The median age of all participants was 43 years, and the median waist circumference was 98.9 cm, with a median BMI was 28.50 kg/m^2^.

Table 1 summarizes the demographic characteristics by the prevalence of asthma attack. Waist circumference was statistically different (*P* < 0.001). The weighted median age was 41 years for the non-asthma attack group and 46 years for the asthma attack group, with significant age variations between the two groups (*P* < 0.001). Compared with 97.3 cm in the non-asthma attack group, the weighted median waist circumference of the asthma attack group was 102.6 cm, indicating a statistically significant difference in waist circumference (*P* < 0.001). Consistently, in the asthma attack group, the BMI was notably elevated compared to the non-asthma attack group (30.4 kg/m^2^ *vs*.27.8 kg/m^2^, *P* < 0.001).

**Table 1:** The demographic characteristics of the study population

	Non-asthma attack *N* = 3968	Asthma attack *N* = 1562	*P*-value
Age (years)	41.0 (28.0, 56.0)	46.0 (32.0, 57.0)	< 0.001
Gender (%)			< 0.001
Female	53.4	68.8	
Male	46.6	31.2	
Race (%)			0.263
Non-Hispanic White	69.2	69.7	
Non-Hispanic Black	12.8	13.4	
Mexican American	5.6	4.2	
Other Hispanic	5.5	5.2	
Other Race—Including Multi-Racial	6.9	7.5	
Education (%)			0.156
Below high school	14.3	14.8	
High School	22.6	19.9	
Above high school	63.1	65.3	
BMI (kg/m^2^)	27.8 (24.0, 33.2)	30.4 (25.2, 36.0)	< 0.001
Waist circumference (cm)	97.3 (85.9, 110.4)	102.6 (89.3, 115.5)	< 0.001
Diabetes (%)			< 0.001
No	90.8	86.5	
Yes	9.2	13.5	
Smoking (%)			0.137
No	51.8	49.0	
Yes	48.2	51.0	
Drinking (%)			0.025
No	90.3	87.8	
Yes	9.7	12.2	

*BMI* Body mass index

### Association of waist circumference with asthma attack

Table 2 shows the association between waist circumference and asthma attack. Analysed as a continuous variable, waist circumference exhibited significant association with asthma attack across the non-adjusted, minimally adjusted, and fully adjusted model, displaying ORs (95% CI) of 1.07 (1.05, 1.09), 1.06 (1.04,1.09), and 1.06 (1.02, 1.10), respectively. After full adjustment for BMI-defined obesity, age, gender, race/ethnicity, education levels, PIR levels, smoking status, and metabolic syndrome, every 5 cm increase in waist circumference exhibited a 1.06 times higher likelihood of asthma attack probability.

**Table 2:** The association between waist circumference and poor asthma control using weighted logistic regression models

	**Non-adjusted model**		**Minimally adjusted model**		**Fully adjusted model**	
	OR (95% CI)	*P*	OR (95% CI)	*P*	OR (95% CI)	*P*
Waist circumference (Per 5 cm)	1.07 (1.05, 1.09)	< 0.001	1.06 (1.04, 1.09)	< 0.001	1.06 (1.02, 1.10)	0.002
Categories						
Q1	*Reference*		*Reference*		*Reference*	
Q2	1.14 (0.90, 1.44)	0.276	1.12 (0.90, 1.40)	0.317	1.08 (0.87, 1.36)	0.482
Q3	1.48 (1.19, 1.85)	< 0.001	1.38 (1.06, 1.79)	0.017	1.20 (0.88, 1.63)	0.238
Q4	1.77 (1.43, 2.19)	< 0.001	1.58 (1.22, 2.03)	< 0.001	1.30 (0.92, 1.83)	0.138

Q1, 57.9–87.8 cm; Q2, 87.8–99.85 cm; Q3, 99.85–113.2 cm; Q4, 113.2–170.5 cm

Minimally adjusted model: We adjusted for age, gender, race/ethnicity, education levels, PIR levels, smoking status, and metabolic syndrome

Fully adjusted model: We adjusted for BMI-defined obesity, age, gender, race/ethnicity, education levels, PIR levels, smoking status, and metabolic syndrome

*OR* Odds ratio, *CI* Confidence interval

### Dose-dependent association between waist circumference and asthma attack

**Fig. 2:**
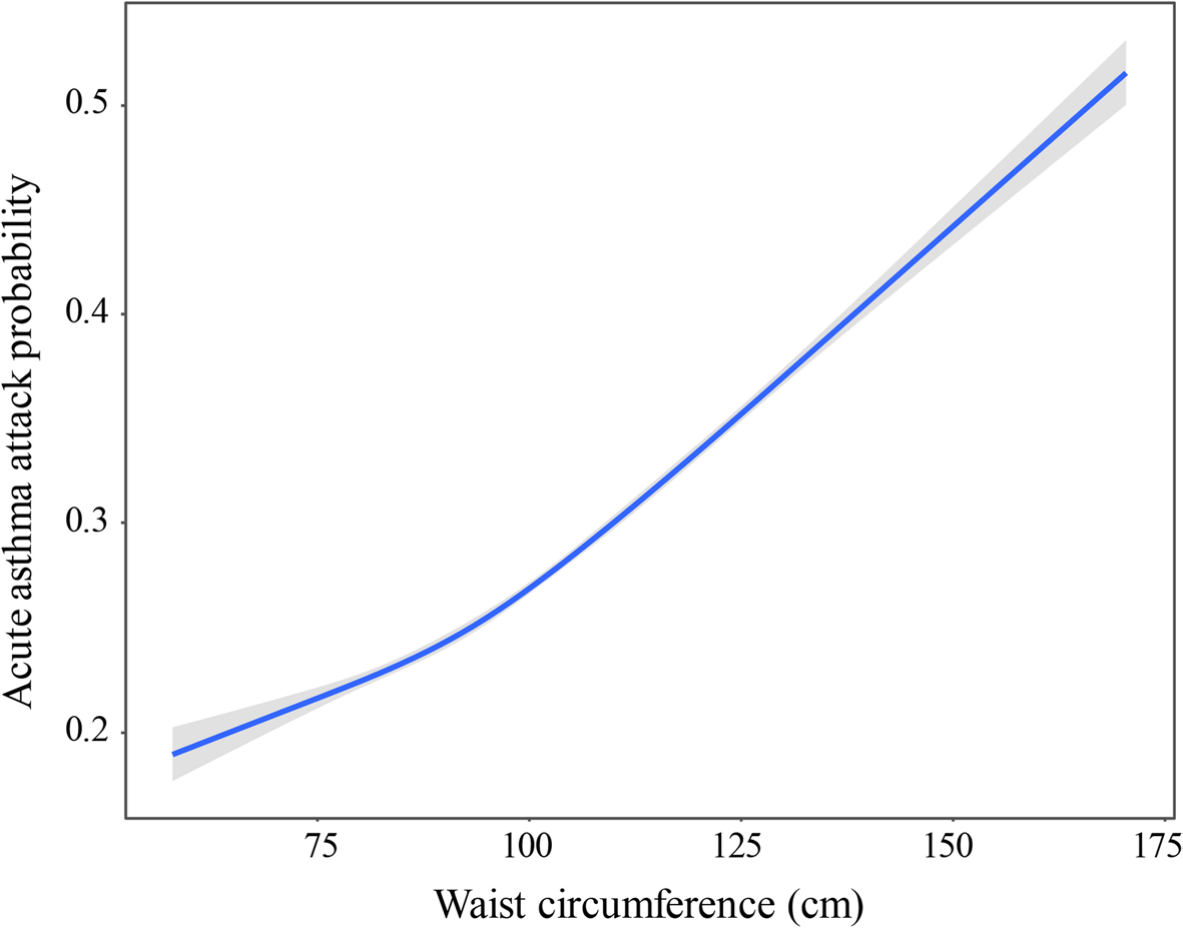
Restricted cubic spline plots of the association between waist circumference and asthma attack probability

We visualized the dose-dependent association between waist circumference and asthma attack probability using weighted restricted cubic splines. As shown in Fig. 2, the risk of asthma attack elevates with the increasing waist circumference when adjusting for BMI, age, gender, race, education levels, and current smoking.

**Fig. 3:**
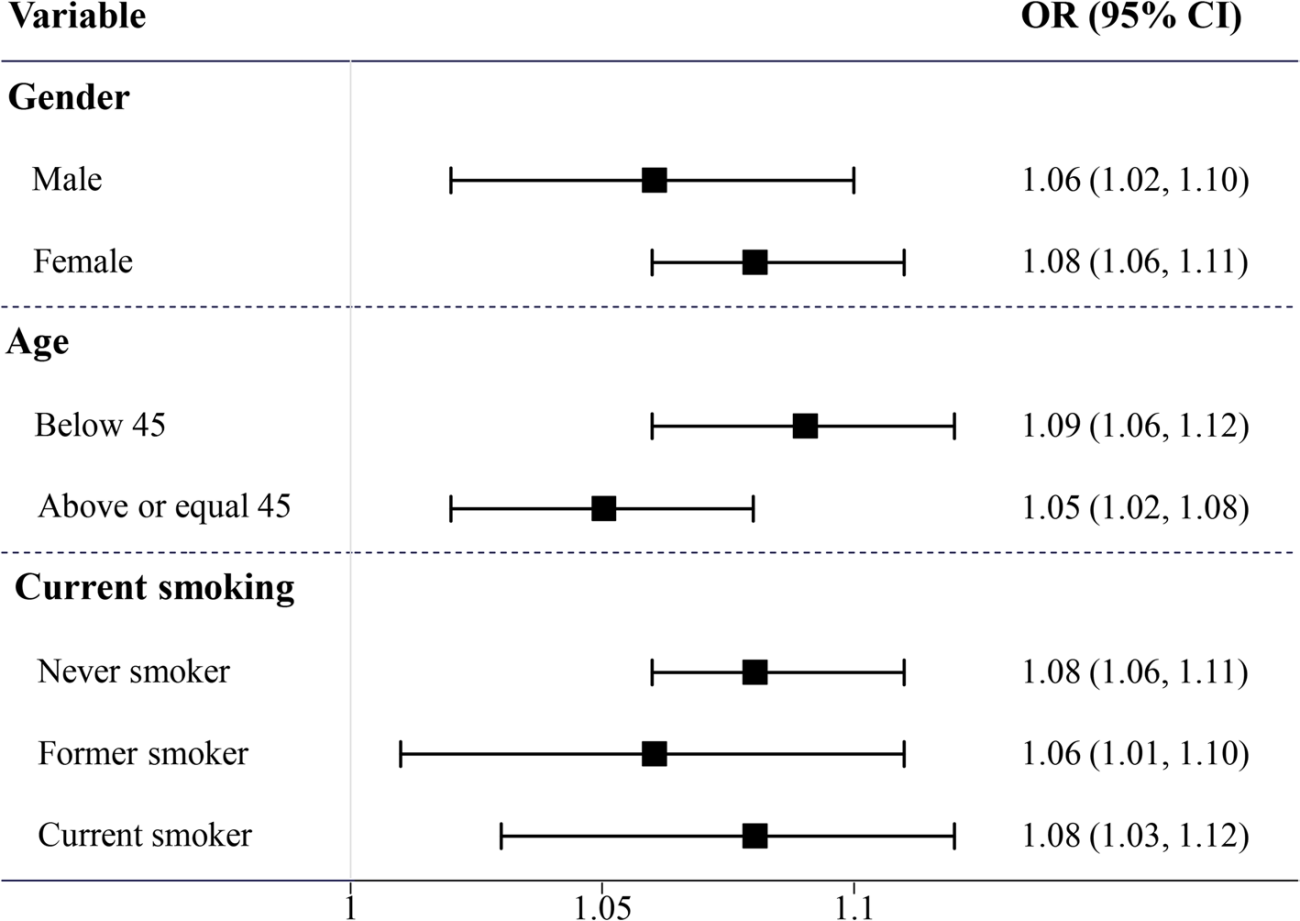
Forest plot of the subgroup analysis of the association between waist circumference and asthma attack probability based on logistic regression analysis across sex (male or female), age (< 45 or ≥ 45 years), and current smoking (not at all, some days or every day). The association was adjusted for waist circumference, age, race, education, and current smoking status. *OR: Odds ratio; CI: Confidence interval*

Illustrated in Fig. 3, the positive association between waist circumference and asthma attack probability remained robust within various subgroups, encompassing gender (male or female), age (< 45 or ≥ 45 years), and smoking (never, former, and current smokers).

### Sensitivity analysis

To further validated the association between waist circumference and adult asthma attack, we employed a more stringent criterion for defining an asthma attack. Specifically, only participants who both experienced an asthma episode and required an emergency room or urgent care center visit due to asthma were classified as having an asthma attack. After adjusting for BMI-defined obesity, age, gender, race/ethnicity, education levels, PIR levels, smoking status, and metabolic syndrome, the weighted logistic regression models demonstrated the strong association between waist circumference and asthma attack (Table 3). An increase of every 5 cm in waist circumference was associated with an OR (with 95% CI) of 1.08 (1.02, 1.15). Moreover, individuals in the highest quartile (Q4) of waist circumference were found to have a 2.12-fold greater likelihood of experiencing asthma attack compared to those in the lowest quartile (Q1).

**Table 3:** The sensitivity analysis association between waist circumference and poor asthma control using weighted logistic regression models

	OR (95% CI)	*P*
Waist circumference (Per 5 cm)	1.08 (1.02, 1.15)	0.009
Categories		
Q1	*Reference*	
Q2	1.44 (0.87, 2.39)	0.158
Q3	1.49 (0.87, 2.58)	0.146
Q4	2.12 (1.09, 4.11)	0.026

Q1, 57.9–87.8 cm; Q2, 87.8–99.85 cm; Q3, 99.85–113.2 cm; Q4, 113.2–170.5 cm

BMI-defined obesity, age, gender, race/ethnicity, education levels, PIR levels, smoking status, and metabolic syndrome were adjusted for

*OR* Odds ratio, *CI* Confidence interval

## Discussion

To explore the cross-sectional association between waist circumference and asthma attack, the investigation included 5,530 participants from the 2003 to 2018 NHANES Survey. When contrasting individuals with asthma attack to those with non- asthma attack, the asthma attack group exhibited notably higher waist circumference compared to the non-asthma attack group. To further explore the impact of waist circumference on asthma attack, we constructed a weighted adjusted logistic regression analysis model to assess the horizontal relationship between waist circumference and asthma attack. When waist circumference was analyzed as a continuous factor (adjusting for BMI-defined obesity, age, sex, race, education, and smoking level), the OR (95%CI) attributed to waist circumference in the NHANES survey was 1.07 (1.03, 1.10). When waist circumference was examined as a four-way categorical variable (adjusting for BMI-defined obesity, age, sex, race, education level, and smoking status), the OR values (95%CI) of the NHANES survey in the high waist circumference group (Q4) compared with the low waist circumference group (Q1) were: 1.54 (1.13, 2.10), cross-sectional data analysis of this study suggests that an increase in waist circumference may increase the risk of asthma attack.

In recent years, many large-scale population cohort studies have put forth the existence of the “obesity paradox,” finding BMI has a J-shaped or U-shaped curve relationship with the risk of illness or death from various diseases [52, 53]. However, chronic respiratory diseases are also influenced by the “obesity paradox,” particularly in the cases of lung cancer and chronic obstructive pulmonary disease [54, 55]. There is a complex association between metabolic disorders, airway inflammation and exacerbation of asthma [56]. It is a prevailing consensus that obesity can lead to a higher prevalence of asthma and an elevated risk of related adverse incidents, so the “obesity paradox” is rarely discussed in the context of asthma development [56]. Obesity has been documented to have a connection with poor asthma control [57–59]. Another study showed that obese individuals (BMI ≥ 28 kg/m^2^) exhibited a considerable frequency of poorly controlled asthma (62.9%), and they faced a 1.31-fold increased risk of having uncontrolled asthma when compared to participants with a normal BMI [13].

There is growing evidence that abdominal obesity has evolved into a more severe problem globally extending beyond the scope of obesity defined solely by BMI [60]. At present, the diagnostic criteria for abdominal obesity are based on the conversion of BMI, ignoring the clinical significance of waist circumference itself and failing to take into account the unique advantages of waist circumference in evaluating the metabolic risk linked to the distribution of abdominal fat [61]. The present measures of abdominal obesity, such as waist circumference, have been reported better to predict some obesity-related diseases [26–33]. It’s advisable to consider measures beyond BMI. when investigating the impact of obesity on self-reported asthma according to a study [62]. A meta-analysis has shown that there is a positive association between asthma and abdominal obesity, as measured by waist circumference [28]. Nonetheless, the relationship between waist circumference and asthma attack remains uncertain. Current clinical practice does not require routine measurement of waist circumference or only recommends additional measurement of waist circumference for overweight or obese people [20, 63, 64]. In 2020, an expert consensus pointed out that the current research on waist circumference and metabolic diseases is not deep enough, and the practical use of waist circumference in clinical settings is inadequate [34]. A suggestion is to incorporate waist circumference as a “vital sign” of routine measurement in clinical assessments to comprehensively assess the metabolic risk of fat distribution [34]. Further investigation was conducted to examine the association between waist circumference and metabolic diseases at different BMI levels [34]. An in-depth understanding of the association between waist circumference and asthma attack is advantageous for enhancing the management of asthma attack risk associated with abdominal obesity and provides the theoretical basis for tertiary prevention of hypertension. Therefore, this study used NHANES to analyze waist circumference as a continuous variable and a categorical variable, respectively, to explore the horizontal association of hypertension.

Research has verified a distinct “asthma-obesity” respiratory metabolic phenotype [65] and the causality between obesity and asthma has been reinforced [56]. Obese patients exhibit a certain type of subclinical chronic inflammation that can cause airway inflammation, reduced lung function, and asthma exacerbations [62]. Due to the variability of asthma, regular monitoring of control levels is essential to assess whether therapy should be maintained or adjusted [5]. Although asthma is incurable, several studies have suggested that accurate diagnosis, appropriate treatment, and ongoing management can lead to better asthma control and improved quality of life for individuals with asthma [66].

As for the measurement of waist circumference, there lacks a universally accepted standard, largely due to the absence of a theoretical basis for selecting specific measurement sites. Currently, three principal sites are predominantly used in both clinical and research contexts: the midpoint between the lowest rib and the iliac crest’s highest point, as recommended by the World Health Organization; the iliac crest’s upper edge, advocated by the National Institutes of Health; and the navel level, frequently selected in many clinical studies [67, 68]. These varied methodologies each capture different dimensions of abdominal fat, possibly leading to discrepancies in correlating waist circumference with health outcomes, such as adult asthma attacks. Given the variability in measurement techniques and the potential influence of sex-specific anatomical and physiological differences in fat distribution, it is crucial for future research and clinical practice guidelines to strive for the standardization of waist circumference measurement. Additionally, investigating whether certain measurement sites are more predictive of health risks in men versus women could provide valuable insights. Such standardization would significantly improve the consistency of research findings and the precision of risk assessment in clinical settings, facilitating more accurate health risk evaluations and tailored interventions.

Our study underscores the critical role of waist circumference measurements in the routine health evaluations of individuals diagnosed with asthma, highlighting its inclusion as an essential aspect of comprehensive health assessments. For public health professionals, the findings emphasize the imperative of advocating and implementing holistic strategies that target obesity and overweight, key contributors to increased waist circumference, as integral components of asthma management and prevention efforts [34]. Given the modifiable nature of waist circumference through dietary adjustments, increased physical activity, and lifestyle modifications, public health initiatives focusing on widespread education and intervention programs hold significant potential in mitigating asthma attacks by promoting healthier living across communities [34]. Furthermore, our results support the integration of waist circumference screening within various community settings, such as schools, workplaces, and health clinics, to enable the early detection of individuals at an elevated risk of asthma attacks. Emphasizing prevention strategies that incorporate weight management and obesity prevention, particularly in environments where they can exert the most considerable influence, is vital. These strategies might include initiatives to foster physical activity, such as community sports events; nutritional education programs aimed at encouraging healthy eating habits; and policy interventions designed to improve access to nutritious foods and recreational facilities. Additionally, recognizing the potentially varied influence of waist circumference on asthma risk among different subgroups, delineated by factors such as gender, age, and smoking status, suggests the benefit of tailored interventions. Addressing the distinct needs and risk profiles of these diverse groups could further refine the efficacy of asthma prevention tactics.

Despite the novel perspectives on waist circumference and asthma attack, limitations should be mentioned. First, following GINA criteria, patients are typically categorized into four groups according to the severity of asthma attacks (intermittent, mild persistent, moderate persistent, or severe persistent) in clinical practice based on spirometry test results, asthma symptoms, and medication use (particularly the doses of inhaled corticosteroids) [69]. However, the NHANES database includes no specific questions about optimal asthma attack criteria recommended by the guidelines. Therefore, the evaluation of asthma attacks in this study is constrained to existing database questions, and we applied grading strategies consistent with the previous studies [37]. Still, the results of this study should be interpreted with caution considering the nonclassical grading strategies on asthma attacks. Second, the definition of asthma exacerbation in this study is based on the questions on whether the participants had an episode of asthma or an asthma attack or had an emergency care visit in the past year. While this approach aligns with methodologies utilized in prior research, it inherently lacks specificity regarding the triggers of asthma exacerbations. Such triggers, including viral infections, allergen exposure, air pollution, and non-adherence to medication, play a crucial role in the clinical definition and understanding of asthma attacks. Our reliance on self-reported data without the granularity to identify specific triggers may not fully encapsulate the complexity of asthma exacerbations as understood in clinical practice. This limitation underscores the necessity of interpreting our findings within the context of these constraints, recognizing that the NHANES data provide a broad, albeit not trigger-specific, perspective on asthma attacks. The results of this study should be interpreted with caution. Third, the research was unable to distinguish the severity of attacks, which can be classified as mild, moderate, severe, and critical. The potential influence of waist circumference on various subtypes of asthma should be further explored. Fourth, although diverse ethnicities exhibit differences in physical activity, dietary habits, genetic variation, lipid metabolism, and vulnerability to respiratory conditions, this research exclusively focused on nationally representative samples from the United States. Since we lack representative data from other regions, such as China and Europe, the applicability of our conclusion to other populations is not definitive. Fifth, although we included multiple confounding factors, it should be noted that several factors associated with asthma exacerbation (such as health insurance coverage, asthma control, asthma severity, use of asthma medication, viral infections, and exposure to indoor or outdoor allergens) were not included in the NHANES survey. The lack of these covariates resulted in the basis of our study when investigating the association between waist circumference and asthma attack.

## Conclusion

Our results suggest that waist circumference is a potential risk factor for asthma attack. The association between higher waist circumference and an increased risk of asthma attacks suggests that waist circumference can be an important marker for identifying adults at higher risk of asthma exacerbations. This insight underscores the necessity for healthcare professionals to incorporate waist circumference measurements into routine assessments of asthma patients, alongside traditional clinical evaluations. The relationship between waist circumference and asthma exacerbations presents a compelling case for integrating weight management strategies into asthma care plans. Such approaches may serve as a dual-purpose intervention, potentially improving asthma outcomes while also addressing the broader public health challenge of obesity.

## Data Availability

All the data were acquired from the National Health and Nutrition Examination Survey (NHANES) database.

## Abbreviations

BMI: Body mass index
CI: Confidence interval
NHANES: National Health and Nutrition Examination Survey
OR: Odds ratio
